# Berberine protects against esophageal mucosal damage in reflux esophagitis by suppressing proinflammatory cytokines

**DOI:** 10.3892/etm.2013.1202

**Published:** 2013-07-04

**Authors:** BYUNG KIL CHOO, SEONG-SOO ROH

**Affiliations:** 1 Department of Crop Agriculture and Life Science, Chonbuk National University, Jeonju 561-756, Republic of Korea; 2 Department of Herbology, College of Korean Medicine, Daegu Haany University, Gyeongsan 712-715, Republic of Korea

**Keywords:** berberine, anti-inflammatory activity, tumor necrosis factor-α, interleukin-1, MCP-1, PAI-1, interleukin-6

## Abstract

This study was performed to investigate the effects of berberine (BB) in a rat model of gastroesophageal reflux disease (GERD), induced by pylorus and forestomach ligation. We evaluated cytotoxicity and proinflammatory biomarkers (nitric oxide, interleukin (IL)-1β and prostaglandin E2) in RAW 264.7 cells *in vitro* and anti-inflammatory effects *in vivo*. A total of 54 Sprague Dawley rats were divided into six groups: intact control rats; reflux esophagitis (RE) control rats; RE rats treated with 20 mg/kg omeprazole and RE rats treated with BB at doses of 20, 40 and 60 mg/kg, respectively. All rats were fasted. RE was induced by pylorus and forestomach ligation one hour subsequent to the oral treatment. Six hours subsequent to the surgery, the rats were sacrificed, blood was collected from the abdominal vein and the esophagus and stomach were dissected. The gastric volume and the pH of the gastric juice were evaluated, prior to the esophagus being cut longitudinally and an inner mucosal area being imaged, to analyze mucosal damage indices. Proinflammatory biomarkers in the serum, including tumor necrosis factor (TNF)-α, IL-1β, IL-6 and monocyte chemoattractant protein (MCP)-1 were analyzed using an enzyme-linked immunosorbent assay (ELISA) kit, while the mRNA expression of TNF-α, IL-1β, IL-6 and plasminogen activator inhibitor (PAI)-1 was analyzed using a quantitative polymerase chain reaction (qPCR). Esophagic tissue damage in the BB groups was dose-dependently decreased compared with that in the RE control group. This result was consistent with significant reductions in the levels of proinflammatory biomarkers in the serum and in the expression of proinflammatory mRNA, specifically, TNF-α, IL-1β, IL-6 and PAI-1. The results suggest that the anti-inflammatory and protective effects of BB may attenuate the severity of RE and prevent esophageal mucosal damage, in addition to validating the use of BB as a pharmacological treatment for esophageal reflux disease.

## Introduction

Reflux esophagitis (RE) is an inflammation of the lower esophagus due to the regurgitation of gastric acid, characterized by a burning pain in the chest (so-called heartburn) and nausea following eating. RE is usually a result of a malfunction of the lower esophageal sphincter (1). In addition, it is associated with an increase in gastric acid secretion and a westernization of lifestyle and diet (i.e. a high-fat diet), as well as a low prevalence of *Helicobacter pylori (H. pylori)* infection.

*H. pylori* is generally accepted as the most important type of bacteria in gastrointestinal disease. However, certain studies have suggested that the increased prevalence of RE following *H. pylori* eradication may be due to the protective role of *H. pylori* infection in patients with RE (2–4).

In humans, prolonged gastroesophageal reflux, including acidic gastric fluid, leads to esophageal mucosal injury, such as bleeding, erythema, erosions and ulcers (5). Medication used to treat RE include antacids, acid blockers, gastric motility agents and surgery. Acid blockers, which comprise histamine type 2 (H2) antagonists and proton pump inhibitors (PPIs), are the most commonly used treatments for RE. H2 antagonists, such as ranitidine and cimetidine, reduce acid production in the stomach, and PPIs, such as omeprazole and esomeprazole, also arrest the production of stomach acid (6). Usually, PPIs are more effective than H2 antagonists (7,8). However, in spite of the marked therapeutic effect, a number of patients have suffered from incidences of relapse and shown incomplete mucosal healing, continued symptoms and complications (9,10). Even with an adequate administration of H2 antagonists and PPIs, 40–60% of patients have suffered from stricture of the esophagus or cancer, instead of recovering from the RE (8).

Previous studies have revealed a number of serious and unusual side-effects resulting from the long-term use of PPIs, such as hypomagnesemia, bowel symptoms and small intestinal bacterial overgrowths (11,12). As a consequence of this fact, there are, at present, safety concerns regarding the long-term use of PPIs, making it necessary to search for effective and safe alternatives (13). Our previous study was carried out to evaluate the potential therapeutic effect of Curculiginis Rhizoma in RE by the suppression of proinflammatory cytokines (14). The reduction of factors that are associated with inflammation is important in the alleviation of RE.

The present study was performed to evaluate the effect of berberine (BB) in an acute model of RE in rats. RE was induced in the rats by pylorus and forestomach ligation, a technique that is considered to create a valuable simple animal model to mimic human RE.

BB, a major natural constituent of the Chinese herb Coptidis Rhizoma, has been shown to exert potent antitumor, anti-inflammatory, antidiarrhea and antidiabetic effects (14–16). BB has been demonstrated to suppress proinflammatory responses through AMP-activated protein kinase (AMPK) activation (17–19) and to inhibit inflammatory cytokines, such as tumor necrosis factor (TNF)-α, interleukin (IL)-1β and IL-6, and inflammatory mediators, such as nitric oxide [NO; produced by inducible nitric oxide synthase (iNOS)] and prostaglandin E2 [PGE2; produced by cyclooxygenase (COX)-2] (20–25).

The anti-inflammatory effects of BB in a rat model of acute RE were investigated by the analysis of gastric secretions, a histological assay of esophageal tissue, an enzyme-linked immunosorbent assay (ELISA) and the analysis of gene expression by quantitative polymerase chain reaction (qPCR). *In vitro* effects in RAW 264.7 cells were also evaluated.

## Materials and methods

### Materials and animals

BB chloride was obtained from Waco Pure Chemical Industries, Ltd (cat. no. 022-05501, lot no. STL2430; Osaka, Japan) and was dissolved in distilled water. Omeprazole was purchased from Sigma-Aldrich (St. Louis, MO, USA) and dissolved in polyethylene glycol (Sigma-Aldrich) at a concentration of 0.1%.

Five-week-old male Sprague-Dawley rats (Central Lab. Animal Inc., Seoul, Korea), weighing 160–180 g, were housed under normal laboratory conditions at 25±1ºC with a controlled 12-h light-dark cycle and maintained on standard rodent chow and tap water. The experimental protocols were performed in accordance with the internationally accepted principles for the use and care of laboratory animals, as stated in the US guidelines (26). When necessary, the rats were deprived of food, although access to water was maintained, 18 h prior to the experiments. All animals were kept in raised mesh-bottom cages to prevent coprophagy. Nine rats were used in each group. The study was approved by the Institutional Review Board (number DHU2012-23).

### Cell culture and chemical treatment

The RAW 264.7 cells were obtained from the American Tissue Culture Collection (Manassas, VA, USA) and were cultured in Dulbecco's modified Eagle’s medium (DMEM) containing 10% fetal bovine serum (FBS) in an atmosphere containing 5% CO_2_. The cells were treated with BB diluted in DMEM with 5% FBS for 24 h, depending on the experimental designs.

### Cell viability assay

The effect of BB on the viability of the cells was estimated using the Cell Counting kit (CCK)-8 (Dojindo Molecular Technologies, Inc., Rockville, MD, USA), in accordance with the manufacturer's instructions. Cells were seeded in a 96-well plate and then incubated with various concentrations (10, 20 and 40 μM) of BB for 24 h. Absorbance was measured with a microplate reader (Thermo Fisher Scientific Inc., Waltham, MA, USA) at 450 nm.

### NO production

RAW 264.7 cells (2.5×10^4^ cells/ml in a 96-well plate) were treated with lipopolysaccharide (LPS; 1 μg/ml) alone or with BB (10, 20 or 40 μM) for 24 h. The culture supernatants were mixed with an equal volume of Griess reagent (Promega Corporation, Madison, WI, USA) and incubated at room temperature for 10 min. Absorbance was measured at 540 nm with a microplate reader. Nitrite levels in the samples were determined by comparisons against a sodium nitrite curve.

### PGE2 and IL-1β production

RAW 264.7 cells were seeded in a 96-well plate at a density of 2.5×10^5^ cells/ml and then treated with LPS (1 μg/ml) alone or with BB (10, 20 or 40 μM) for 24 h. The PGE2 concentration in the culture supernatants was quantified using a competitive enzyme immunoassay kit (R&D Systems Inc., Minneapolis, MN, USA) in accordance with the manufacturer's instructions. IL-1β levels were determined using a commercially available ELISA kit (R&D Systems Inc.), in accordance with the manufacturer's instructions. The ELISA was performed in 96-well polystyrene microplates with a specific monoclonal antibody coating. Absorbance was measured at 540 nm in a microplate reader.

### Acute RE induction

All 54 rats were starved of food for 18 h prior to the RE induction surgery; however, free access to water was maintained. The rats were anesthetized with an intraperitoneal injection of 0.75 mg/kg Zoletil^®^ (Virbac S.A., Carros, France). A midline laparotomy was performed to expose the stomach, prior to the pylorus and the transitional junction between the forestomach and the corpus being exposed and subsequently ligated with 2-0 silk thread (only sham control rats were not ligated). The vagus nerves were left intact. Following surgery, the 54 rats were divided into six groups of nine rats each. In the intact control group and the RE control group, no further treatment was performed in addition to the previously mentioned surgical procedure. However, the rats in the omeprazole group were additionally treated with 20 mg/kg omeprazole 1 h prior to surgery and the rats in the BB groups were treated with 20, 40 and 60 mg/kg BB, respectively, 1 h prior to abdominal surgery.

### Analysis of gastric secretions

After sacrifice, each rat stomach was washed with 1 ml phosphate-buffered saline (PBS) with a 1,000 μl micropipette and the gastric contents were collected. In addition, the volume of gastric juice was examined. The pH of the collected gastric juice was measured using a pH meter (EcoMet; iSTEK Co., Seoul, South Korea).

### Effect of BB on cytokine levels in the serum

In order to study the effect of BB on cytokine levels in the serum, immediately following the termination of the experiment, venous blood samples were drawn from the abdominal vein, placed into vials and used for the determination of plasma TNF-α, IL-1β, IL-6 and monocyte chemoattractant protein (MCP)-1 levels. Blood samples were collected and centrifuged at 1,800 × g for 15 min at a temperature of 15ºC, prior to the plasma being collected using a micropipette and stored at −80ºC until the ELISA was performed.

The serum levels of the proinflammatory cytokines, TNF-α, IL-1β and IL-6, and the chemokine, MCP-1, were evaluated with a Multi-Analyte ELISArray^®^ kit (Millipore (Rockford, IL, USA), in accordance with the manufacturer's instructions. The color intensity of the reaction was estimated using a Luminex luminometer (Awareness Technology Inc., Palm City, FL, USA) at 490 nm.

### Effect of BB on TNF-α, IL-1β, IL-6 and plasminogen activator inhibitor (PAI)-1 mRNA transcript expression level by qPCR

Total RNA was extracted from the intestinal graft from the esophagus using TRIzol reagent (Invitrogen Life Technologies, Inc., Grand Island, NY, USA), in accordance with the manufacturer's instructions. RNA content was measured by 260/280 UV spectrophotometry. qPCR analysis, using a SYBR-Green PCR kit, was conducted using glyceraldehyde 3-phosphate dehydrogenase (GAPDH), IL-6, TNF-α, IL-1β and PAI-1 primers (Table I), as described previously (1). The expression of all transcripts was normalized to GAPDH levels. qPCR analysis was conducted using an ABI PRISM^®^ 7000 Sequence Detection System (Applied Biosystems, Foster City, CA, USA), in accordance with the manufacturer's instructions. The thermal cycling conditions were: 10 min at 95ºC to activate the Amplitaq Gold^®^ DNA polymerase, followed by 40 cycles of 95ºC for 15 sec and 60ºC for 1 min with the ABI PRISM^®^ 7000 Sequence Detection System (Applied Biosystems). Using the manufacturer's software, qPCR data were plotted as the fluorescence signal versus the cycle number. The cycle threshold was defined as the cycle number at which the fluorescence signal crossed the threshold.

The expression of each gene was normalized to GAPDH mRNA content and calculated relative to the control using the comparative cycle threshold method.

### Determination of gross and microscopic esophageal mucosal damage

Rats were sacrificed and the entire esophagus was removed, prior to a lengthwise incision being made in the esophagus using scissors. Following this, the esophagus was gently rinsed with 0.9% NaCl and an image was captured using a digital camera (Sony, Tokyo, Japan). Examination of gross mucosal injury was conducted using the i-Solution Lite software program (Innerview Co., Sungnam, South Korea) and a gross lesion index was applied as follows: 0, appeared as normal glistening mucosa; 1, edematous mucosa with focal hemorrhage spots; 2, multiple erosions with hematins attached; 3, linear ulcerations with yellowish exudates and 4, coalesced ulcerations. The gross esophageal mucosa protecting ratio was calculated as follows: Esophageal mucosa protecting ratio (%)=[total area of esophagus (mm^2^)-width of area with esophageal mucosal injury (mm^2^)]/width of total area of esophagus (mm^2^) ×100.

For microscopic evaluation, the opened esophagus was cut to isolate the middle segment. This segment was embedded in paraffin, cut into 2-μm sections and stained using hematoxylin and eosin (H&E) for microscopic evaluation. The stained slices were subsequently observed under an optical microscope, analyzed using the i-Solution Lite software program (Innerview Co.) and assessed using the three-part histological activity index as follows: i) extent of esophageal ulcers: 0, none; 1, erosion; 2, multiple erosions; 3, ulceration and 4, large, excavated ulcer; ii) degree of inflammation: 0, none; 1, mild; 2, moderate; 3, severe and 4, absent; iii) damage to the mucosa: 0, 0–10%; 1, 10–30%; 2, 30–60% and 3, 60–100%.

### Statistical analysis

Results are expressed as the mean ± standard deviation. Statistical analysis was performed using analysis of variance (ANOVA) and two-way ANOVA tests with a Tukey post hoc test where appropriate. Data are expressed as the mean ± standard deviation of each group. A Student's t-test was also used for statistical analysis. P<0.05, P<0.01 and P<0.001 were considered to indicate statistically significant differences.

## Results

### Cytotoxicity and anti-inflammatory activity of BB in vitro

**Figure 1:**
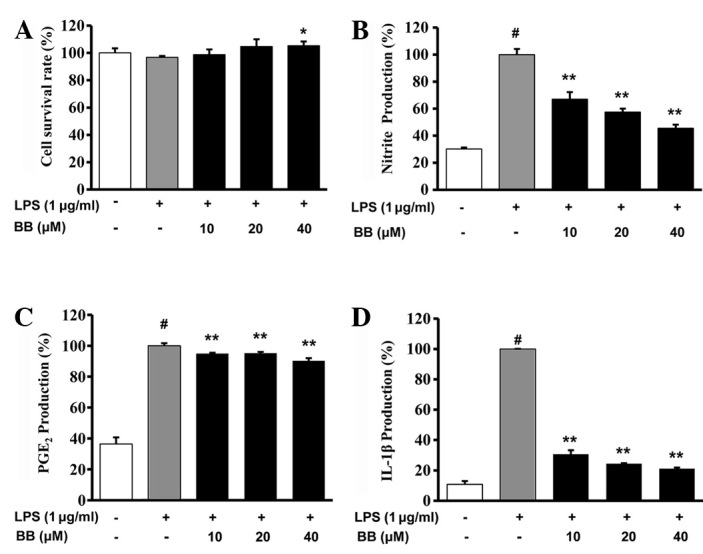
Effects of berberine (BB) on cell proliferation and the lipopolysaccharide (LPS)-induced release of nitric oxide (NO), prostaglandin E2 (PGE2) and interleukin (IL)-1β in RAW 264.7 cells. (A) A Cell Counting kit (CCK)-8 assay was used to evaluate the proliferation of RAW 264.7 cells treated for 24 h with LPS alone or with LPS and BB. (B) The effect of BB on LPS-induced NO production was estimated as described in Materials and methods. (C) PGE2 production was measured in RAW 264.7 cells treated for 24 h with LPS alone or with LPS and BB, using an enzyme immunoassay kit. (D) Release of IL-1β was also measured using an enzyme immunoassay kit. Each data point represents the mean ± standard deviation of three independent experiments. ^#^P<0.05 compared to control; ^*^P<0.05; ^**^P<0.01 compared with LPS alone.

To examine the effect of BB on cell growth in RAW 264.7 cell, the cells were treated with various concentrations (10, 20 and 40 μM) of BB for 24 h. It was observed that BB did not affect normal cell growth (Fig. 1A). Thus, in the following experiments, the effects of BB at all concentrations were studied on cells with a normal growth status. Inflammatory leukocytes are stimulated by LPS, leading to the release of inflammatory mediators of NO and PGE2. In the evaluation of the anti-inflammatory effect of BB on RAW 264.7 cells, it was observed that BB inhibited the LPS-induced elevation of NO production; at concentrations of 10, 20 and 40 μM, BB inhibited the production of NO by RAW 264.7 cells in a concentration-dependent manner. Furthermore, the amount of PGE2 released by the BB-treated cells was reduced in comparison with that by the LPS-treated control cells (Fig. 1C). The level of IL-1β production by the activated RAW 264.7 cells was significantly increased compared with that of the normal control cells (P<0.05); however, the IL-1β production of the cells treated with each concentration of BB was significantly reduced compared with that of the LPS-treated control group (P<0.01, Fig. 1D).

### Effects on gross mucosal damage

**Figure 2:**
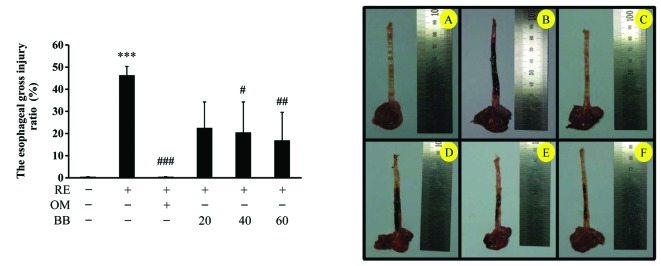
Effects of berberine (BB) on gross esophageal damage in rats with reflux esophagitis (RE). The rat esophagus was removed immediately subsequent to sacrifice and cut in the longitudinal direction from the gastroesophageal junction to the pharynx. The inner mucous was washed away with phosphate-buffered saline (PBS). The dissected esophagus was laid out on paper and photographic images captured with an optical digital camera, and the damage was analyzed using the i-Solution Lite software program (Innerview Co., Sungnam, South Korea). (A) Normal intact rat esophagus; (B) esophagus from rat with surgically induced acute RE treated with distilled water; (C) esophagus with induced acute RE treated with 20 mg/kg omeprazole (OM); (D–F) esophagi with induced acute RE treated with (D) 20, (E) 40 and (F) 60 mg/kg BB. Values are expressed as the mean ± standard deviation. ^***^P<0.001 compared with the intact rat group; ^#^P<0.05 and ^##^P<0.01 compared with the RE control rat group. RE, control rat with pylorus and forestomach ligation treated with distilled water. OM, positive control rat with pylorus and forestomach ligation treated with omeprazole (20 mg/kg). BB, rat with pylorus and forestomach ligation treated with BB (20, 40 and 60 mg/kg, respectively).

In the normal intact group, no lesion damage or mucosal injury, such as hyperemia, multiple erosions with hematins attached, coalesced ulcerations or serosanginous exudates were observed (Fig. 2A). However, a severe longitudinal lesion with hyperemia, multiple erosions with hematins attached, coalesced ulcerations and serosanginous exudates were observed in the esophagi of the RE control group (Fig. 2B). The pathological area of the esophagi of the RE control group was grossly increased compared with that in the intact group. However, the positive control group treated with 20 mg/kg omeprazole had less damage than the RE control group (Fig. 2C). The gastric injury of each BB-treated group comprised only scattered erosions or mild hemorrhagic spots with whitish exudates scattered along the esophagus (Fig. 2D–F).

The gross injury index of the RE control was 46.4±3.9%, whereas the index of the positive control treated with omeprazole was 0.5±0%, and the indices of the groups treated with 20, 40 and 60 mg/kg BB were 22.6±11.7, 20.5±13.8 and 16.9±12.6%, respectively. Therefore, the gross injury indices of the BB groups were significantly dose-dependently decreased compared with that of the RE control (Fig. 2).

**Figure 3:**
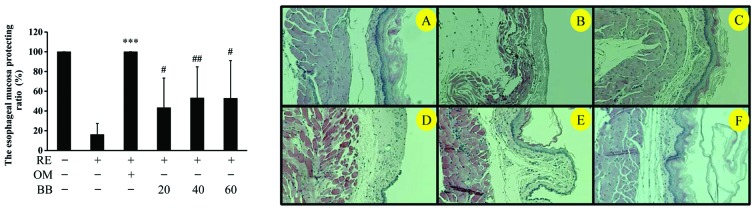
Effect of berberine (BB) on the histology of the esophageal mucosa. The rat esophagus was removed immediately subsequent to sacrifice and cut in the longitudinal direction from the gastroesophageal junction to the pharynx. The middle portion of the esophagus was collected and sections of the esophagus were stained with hematoxylin and eosin. Images were captured using an optical digital camera and analyzed with the i-Solution Lite software program (Innerview Co., Sungnam, South Korea). (A) Normal intact rat; (B) control rat with reflux esophagitis (RE) induced by pylorus and forestomach ligation treated with distilled water; (C) positive control rat with pylorus and forestomach ligation treated with omeprazole (OM; 20 mg/kg); (D–F) rat with pylorus and forestomach ligation treated with (D) 20, (E) 40 and (F) 60 mg/kg BB. Magnification, × 100. ^***^P<0.001 compared with the intact rat group; ^#^P<0.05 and ^##^P<0.01 compared with the RE control rat group.

The macroscopic observations of the rats with RE with and without pretreatment with BB are shown in Fig. 3. Mucosal ulceration was markedly suppressed in the rats pretreated with BB (Fig. 3).

### Effect on gastric volume and gastric juice pH

**Figure 4:**
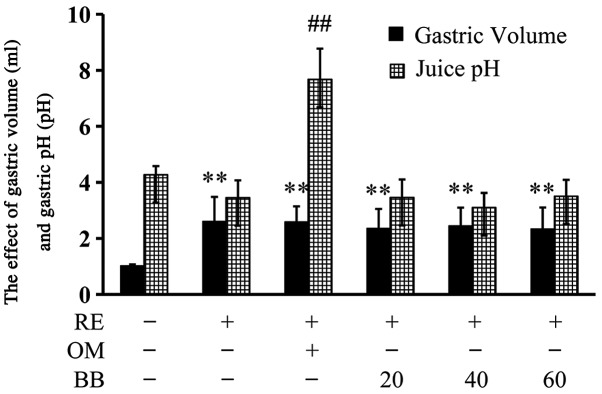
Effect of berberine (BB) on gastric volume and gastric juice pH in rats with reflux esophagitis (RE). The rat stomach was removed immediately subsequent to sacrifice and washed with phosphate-buffered saline (PBS) to collect the gastric contents. The gastric contents were centrifuged at 1,800 × g for 5 min and their volumes were measured. The pH of the collected gastric juice was also measured using a pH meter. Values are expressed as the mean ± standard deviation of nine rats. ^**^P<0.01 compared with normal, intact rats.^##^P<0.01 compared with RE rats. RE, rat with pylorus and forestomach ligation, treated with distilled water; OM, rat with pylorus and forestomach ligation, treated with omeprazole (20 mg/kg). BB, rat with pylorus and forestomach ligation, treated with BB (20, 40 or 60 mg/kg).

The gastric volume of the normal intact group was 1.03±0.04 ml (containing 1 ml PBS). However, the gastric volume of the RE control group was significantly increased compared with that of the intact group (2.61±0.8 ml; P<0.01). The gastric volumes of the positive control group treated with 20 mg/kg omeprazole (2.6±0.8 ml) and the RE groups pretreated with 20, 40 and 60 mg/kg BB (2.4±0.7, 2.45±0.6 and 2.3±0.8 ml, respectively) were not significantly different compared with that of the RE control group; however, they were significantly higher than that of the intact control group (P<0.01 for all; Fig. 4).

The administration of omeprazole (20 mg/kg) significantly increased the gastric acid pH in the RE rats (P<0.01). However, the gastric acid pH values of the groups treated with BB were not significantly different from those of the RE group (Fig. 4).

### Effects on serum TNF-α, IL-1β, IL-6 and MCP-1 levels

To examine the effects of BB on proinflammatory cytokines (TNF-α, IL-1β and IL-6) and a chemokine (MCP-1) in surgically induced acute RE, cytokine levels in the serum were analyzed.

**Figure 5:**
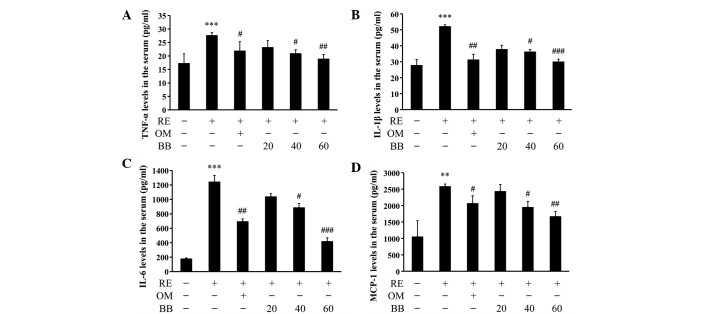
Effect of berberine (BB) on serum levels of tumor necrosis factor (TNF)-α, interleukin (IL)-1β, IL-6 and monocyte chemoattractant protein (MCP)-1. Whole blood was collected from the abdominal vein with a 5 ml syringe, at the time of sacrifice. Collected blood was centrifuged at 1,800 × g for 15 min to collect the serum. Serum levels of the proinflammatory biomarkers (A) TNF-α, (B) IL-1β, (C) IL-6 and (D) MCP-1 were evaluated with the Multi-Analyte ELISArray® Kit (Millipore, Rockford, IL, USA) in accordance with the manufacturer's instructions. Color intensity of the reaction was estimated using the Luminex luminometer(Awareness Technology Inc., Palm City, FL, USA) at 490 nm. Values are expressed as the mean ± standard deviation. ^***^P<0.001 compared with the intact rat group; ^#^P<0.05, ^##^P<0.01 and ^###^P<0.001 compared with the reflux esophagitis (RE) control rat group. RE, control rat with pylorus and forestomach ligation treated with distilled water; OM, positive control rat with pylorus and forestomach ligation treated with omeprazole (20 mg/kg). BB, rat with pylorus and forestomach ligation treated with BB (20, 40 and 60 mg/kg, respectively).

The serum level of TNF-α was 17.3±3.5 pg/ml in intact control rats. However, in the RE rats there was an increase in TNF-α production (27.6±1.0 pg/ml). Treatment of the RE rats with omeprazole (21.9±3.3 pg/ml, P<0.05), 40 mg/kg BB (20.9±1.4 pg/ml, P<0.05) and 60 mg/kg BB (18.9±1.6 pg/ml, P<0.01) significantly inhibited this increase (Fig. 5A).

The serum IL-1β level was 52.1±6.4 pg/ml in the RE control rats, which was significantly higher than the levels in the rats treated with omeprazole (31.3±8.2 pg/ml, P<0.01), 40 mg/kg BB (36.2±9.6 pg/ml, P<0.05) and 60 mg/kg BB (30±11 pg/ml, P<0.001), indicating that these treatments significantly inhibited the increase in IL-1β level following RE induction (Fig. 5B).

The serum IL-6 level of the normal intact group was 178.7±11.5 pg/ml. However, surgically inducing acute RE in the rats resulted in an increase in IL-6 production (1,243±89.5 pg/ml, P<0.001) compared with the control group. Rats treated with 20 mg/kg omeprazole (694.4±35.4 pg/ml), 40 mg/kg BB and 60 mg/kg BB demonstrated significantly lower serum IL-6 levels than the RE control group (P<0.01, P<0.05, and P<0.001, respectively; Fig. 5C).

In the normal intact group, the MCP-1 level was 1052.2±488 pg/ml. However, RE induction in the rats resulted in an increase in the level of MCP-1 production (2,583.5±71.8 pg/ml). The rats treated with 20 mg/kg omeprazole (2,066.7±232.1 pg/ml), 40 mg/kg BB (1,950.4±175.4 pg/ml) and 60 mg/kg BB (1,669.4±150.1 pg/ml) had significantly decreased MCP-1 levels compared with those of the RE control group (P<0.05 for omeprazole and 40 mg/kg BB and P<0.01 for 60 mg/kg BB, Fig. 5D).

### Effects on TNF-α, IL-1β, IL-6 and PAI-1 mRNA expression, analyzed using qPCR

**Figure 6:**
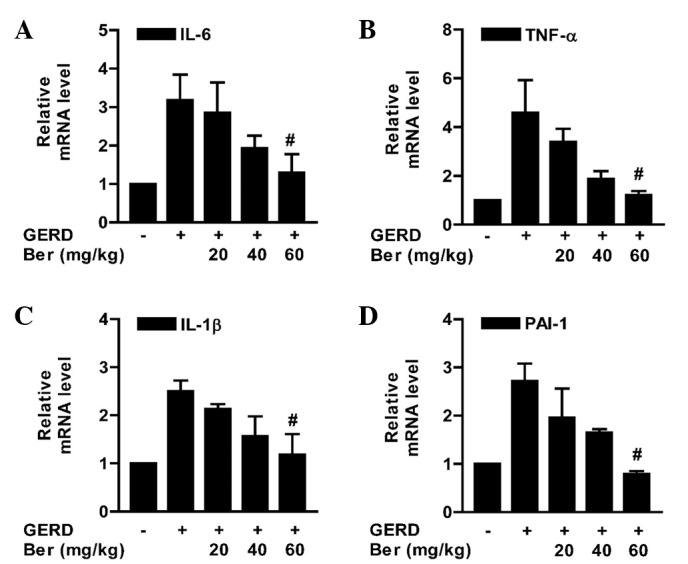
Effect of berberine (Ber) on tumor necrosis factor (TNF)-α, interleukin IL-1β, IL-6 and plasminogen activator inhibitor (PAI)-1 mRNA expression in the esophagus. The rat esophagus was removed immediately subsequent to sacrifice and cut in the longitudinal direction from the gastroesophageal junction to the pharynx. Expression of (A) TNF-α, (B) IL-1β, (C) IL-6 and (D) PAI-1 mRNA was quantified by quantitative polymerase chain reaction (qPCR) in intact animals, reflux esophagitis (RE) control rats and RE rats pretreated orally with 20, 40 or 60 mg/kg berberine. RE was induced by pylorus and forestomach ligation. RE control rats were treated with distilled water. Values are expressed as the mean ± standard deviation. ^#^P<0.001 compared with the RE control rat group. GERD, gastroesophageal reflux disease.

As shown in Fig. 6, the expression levels of TNF-α, IL-1β, IL-6 and PAI-1 mRNA were low in the intact esophageal mucosa. However, mRNA expression levels in the RE control were significantly increased, due to the inflammatory reaction in the esophagus. The expression levels of TNF-α, IL-1β, IL-6 and PAI-1 mRNA in the rats pretreated with a concentration of 60 mg/kg BB were significantly decreased compared with those of the RE control group (P<0.05, Fig. 6).

### Histological analysis of esophageal mucosa

Esophageal tissue stained with H&E revealed no microscopic mucosal changes in the intact rat (Fig. 3A). The normal esophagus exhibited a thin epithelial layer with squamous cells and inflammatory cells were not observed in the submucosal layers. By contrast, 6 h subsequent to the induction of RE, the RE group rats developed large coalesced longitudinal ulcers in the lower and middle sections of the esophagus. Mucosal damage and hyperemia of the epithelial layers and edema and hemorrhage in the mucosa and submucosa were observed in the RE control animals (Fig. 3B). Furthermore, the mucosal layers were damaged by gastric acid. By contrast, esophageal damage, edema, neutrophil infiltration and gastric hemorrhage were not observed in the rats treated with omeprazole (Fig. 3C) and the RE rats treated with 40 and 60 mg/kg BB (Fig. 3E and F, respectively) also showed less severe pathological changes.

The histological activity indices were significantly reduced in the rats pretreated with omeprazole and BB (Table II).

## Discussion

The present study demonstrated that the administration of BB significantly inhibited gastric acid-induced esophageal damage. A number of herbal therapies have been proposed for the treatment of RE; however, the efficacy of BB as a treatment for RE has not, to the best of our knowledge, been investigated.

The pathogenesis of RE is associated with oxidative stress, inflammation and apoptosis. A number of studies have demonstrated the suppressive effect of BB on inflammation (27–29). The proinflammatory cytokines, TNF-α, IL-1β and IL-6, are important in the response to microbial infection or tissue damage. BB treatment has been reported to significantly downregulate the expression of proinflammatory genes, such as TNF-α, IL-1β, IL-6 and MCP-1 (30).

In the present study, we investigated whether BB inhibited the inflammation associated with RE. The suppressive effects of BB on the production of inflammatory mediators in LPS-stimulated RAW 264.7 cells and rat serum were observed.

Two of the major cytokines are TNF-α and IL-1. TNF-α induces a number of physiological effects, including septic shock, inflammation and cytotoxicity. TNF-α is also known as cachectin, due to the fact that it mediates fever and cachexia, and is responsible for the numerous detrimental effects associated with bacterial sepsis, rheumatoid arthritis and Crohn's disease. TNF-α is released by monocytes and macrophages in response to various stimuli including bacterial LPS, which is a principal mediator of the deleterious effects of endotoxin (31). IL-1 is an inflammatory cytokine that is released in response to infection or cell injury by cells of the innate immune system, such as macrophages. Keratinocytes store and release IL-1 following wounding of the skin, rapidly signaling to the surrounding cells that the external barrier has been damaged. IL-1β is important for the initiation and enhancement of the inflammatory response (32,33).

IL-6 is produced in various cells, such as fibroblasts, monocytes, T cells, B cells, microglia, endothelial cells, neurons and astrocytes (34). IL-6, originally identified as a B cell differentiation factor, and synthesized in response to IL-1β, has a critical role in the host reaction to inflammation, inducing the synthesis of acute inflammatory proteins (35). IL-1β and IL-6 are involved in the acute response phase of the immune response (36). IL-6 is a key regulator of cell growth, survival and differentiation, and, as such, is involved in a variety of biological responses, including the immune response, inflammation, hematopoiesis and oncogenesis.

MCP-1 is a member of the C-C chemokine family, and possesses inflammatory properties (37). MCP-1 is important in the recruitment and activation of leukocytes during acute inflammation (38). MCP-1 upregulation is associated with macrophage recruitment, angiogenesis and survival in human breast cancer.

Elizabeth *et al*(39) demonstrated that PGE2 regulated the production of PAI-1 in primary cultures of rat calvarial osteoblasts. PAI-1 production is increased by TNF-α (40) and IL-1 (41). It has been revealed that the induction and transcriptional regulation of the PAI-1 gene may be mediated by cytokines and inflammatory mediators, such as endotoxins, IL-1, transforming growth factor (TGF)-β, insulin, TNF-α, hepatocyte growth factor (HGF) and phorbol 12-myristate 13-acetate (42).

BB is known to inhibit inflammatory cytokines, such as TNF-α, IL-1β and IL-6, and inflammatory mediators, such as NO (produced by iNOS) and PGE2 (produced by COX-2) (20–25,27–29). In the present study, the gastric volume and the pH of the gastric juice in the RE rats were not significantly altered following treatment with BB, so the BB-treated rats were stimulated by gastric acid to the same extent as the RE control rats. However, the gross esophageal lesions and histological indications of mucosal damage in the BB-treated rats were significantly reduced compared with those in the control RE rats, with regard to the extent of esophageal ulcers, the degree of inflammation, the damage to the mucosa and the survival ratio of the mucosal layer.

In conclusion, the present results indicate that BB suppresses inflammation of the esophagus. In support of our hypothesis, NO, PGE2 and IL-1β production levels were significantly diminished *in vitro* in RAW 264.7 cells that had been stimulated with LPS. Furthermore, serum levels of the inflammatory biomarkers, TNF-α, IL-1β, IL-6 and MCP-1, were significantly reduced *in vivo* in rats treated with BB, compared with the levels in untreated RE rats. TNF-α, IL-1β, IL-6 and PAI-1 mRNA expression levels in esophageal tissue was also significantly reduced compared with the levels in the untreated RE control rats.

## Figures and Tables

**Table I:** Sequences of primers.

Gene	Primer direction	Sequence
IL-1β	Forward	5′-CAC CTC TCA AGC AGA GCA CAG-3′
	Reverse	5′-GGG TTC CAT GGT GAA GTC AAC-3′
TNF-α	Forward	5′-CCA GGA GAA AGT CAG CCT CCT-3′
	Reverse	5′-TCA TAC CAG GGC TTG AGC TCA-3′
IL-6	Forward	5′-CGAAAGTCAACTCCATCTGCC-3′
	Reverse	5′-GGCAACTGGCTGGAAGTCTCT-′3
PAI-1	Forward	5′-CCGATGGGCTCGAGTATGA-3′
	Reverse	5′-TTGTCTGATGAGTTCAGCATCCA-3′
GAPDH	Forward	5′-ATGGCACAGTCAAGGCTGAGA-3′
	Reverse	5′-CGCTCCTGGAAGATGGTGAT-3′

IL, interleukin; TNF, tumor necrosis factor, PAI, plasminogen activator inhibitor; GAPDH, glyceraldehyde 3-phosphate dehydrogenase.

**Table II:** Histological activity index in esophageal tissue assessed by hematoxylin and eosin staining.

Score			
	
Group	Extent of esophageal ulcers	Degree of inflammation	Damage to mucosa
Intact	0	0	0
Reflux esophagitis	3.33±0.71^a^	2.78±0.67^a^	3^a^
Omeprazole	0.11±0.33^d^	0^d^	0^d^
Berberine 20 mg/kg	2.83±0.75	2.33±0.51	2.16±1.1
Berberine 40 mg/kg	2.50±0.54^c^	1.33±0.81^b^	2.00±1.3^b^
Berberine 60 mg/kg	1.70±0.51^d^	1.00±0.63^d^	1.10±1.0^c^

Data are presented as the mean ± standard deviation.

a P<0.001 compared with the intact group;

b P<0.05,

c P<0.01,

d P<0.001 compared with control rats with induced reflux esophagitis. The omeprazole group had induced reflux esophagitis and were treated with 20 mg/kg omeprazole; the berberine groups had induced reflux esophagitis and were treated with 20, 40 and 60 mg/kg berberine, respectively.
